# Monitoring the Switching from Base-on to Base-off Forms of Vitamin B_12_ by Natural and Magnetic Circular Dichroism Spectroscopies

**DOI:** 10.1021/acs.analchem.5c07584

**Published:** 2026-01-30

**Authors:** Ewa Machalska, Giuseppe Mazzeo, Aleksandra J. Wierzba, Jakub Dybaś, Joanna E. Rode, Sergio Abbate, Dorota Gryko, Malgorzata Baranska, Giovanna Longhi, Marco Fusè

**Affiliations:** † Department of Molecular and Translational Medicine, Università di Brescia, Via Europa 11, 25123 Brescia, Italy; ‡ Jagiellonian Centre for Experimental Therapeutics (JCET), 37799 Jagiellonian University, Bobrzynskiego 14, 30-348 Krakow, Poland; § Institute of Organic Chemistry, Polish Academy of Sciences, Kasprzaka 44/52, 01-224 Warsaw, Poland; ∥ Laboratory for Spectroscopy, Molecular Modeling and Structure Determination, 86904 Institute of Nuclear Chemistry and Technology, Dorodna 16, 03-195 Warsaw, Poland; ⊥ Istituto Nazionale di Ottica CNR, Unità di Brescia, Via Branze 45, 25123 Brescia, Italy; # Faculty of Chemistry, 37799 Jagiellonian University, Gronostajowa 2, 30-387 Krakow, Poland

## Abstract

This work demonstrates that an approach which makes use of magnetic circular dichroism (MCD) together with electronic circular dichroism (ECD) brings one to a rapid, sensitive, nondestructive, and inexpensive determination of the electronic structure of diamagnetic, chiral, and flexible molecular systems. The subject of this study is cobalamins (Cbls), including vitamin B_12_, the unique and intricate structure of which determines their selective and strong protein binding. Their existence in two forms (base-on and base-off) not only causes significant structural changes but also influences the reactivity of B_12_ derivatives in biologically important organometallic reactions. Therefore, recognizing the Cbl forms and understanding how they switch between them is essential. Notably, this study is the first to show that combining MCD and ECD, supported by quantum mechanics calculations, allows differentiation between base-on and base-off Cbls in aqueous environment at pH 7.4 and in acidic conditions, respectively. Furthermore, these techniques are sensitive to Cbl modifications at the *meso* position of the corrin macrocycle or in the axial upper ligands.

## Graphical abstract

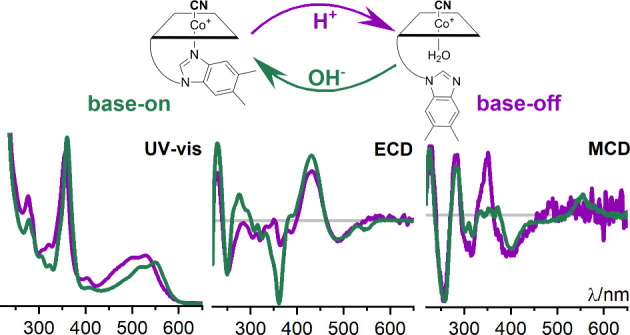

## Introduction

Cobalamins (Cbls), including vitamin B_12_ (**Cbl-1**), are biologically important and structurally relevant coordination complexes. These compounds have been studied regarding their structure,^1–9^ bonding properties,^10–16^ and reactivity,^17,18^ both as coenzymes within apo-proteins and as individual molecules under various nonbiological conditions involving different solvents,^19,20^ light exposure,^21^ and concentrations.^5,19,21^ The most thoroughly investigated Cbl systems through spectroscopy and other chemico-physical methods are **Cbl-1** and B_12_-cofactors.^7,22^ Recently, synthetic Cbls (Figure 1) have attracted increasing interest due to their potential applications in drug delivery,^23–26^ as antivitamins,^27–32^ and ligands for riboswitches.^33–35^ However, their structural characterization remains incomplete; for instance, the electronic and magnetic properties of these compounds have yet to be fully elucidated.

**Figure 1:**
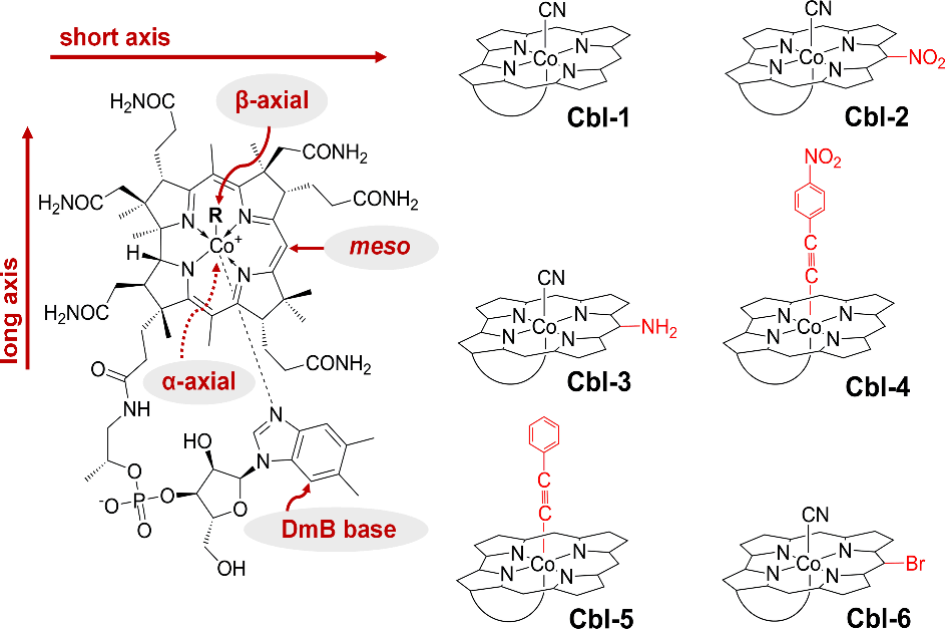
Molecular structures of the studied Cbls in base-on form. Chemical modifications in **Cbl-2** to **Cbl-6** compared to the native **Cbl-1** structure are highlighted in red.

Notably, the ability of Cbls to act as molecular switches is of fundamental significance. At physiological pH, most Cbls predominantly exist in the six-coordinate base-on state,^36,37^ in which the dimethylbenzimidazole base (DmB) is bound to the cobalt atom of the corrin macrocycle (Figure 1). However, Cbls can convert to their protonated base-off form, in which a histidine residue replaces the DmB moiety. This switch occurs in several B_12_-dependent enzymes,^38^ including human enzymes such as methylmalonyl Co-A mutase^39^ and methionine synthase.^40^ Additionally, under some conditions, a water molecule can occupy the DmB site (top part of Figure 3, *vide infra*).^7^ The base-off form with a coordinated water molecule is common in solutions at low pH (1–2),^41–43^ and in specific transport proteins,^44^ including some methyltransferase proteins.^45^

Due to the relevance of the Cbl switch between base-on and base-off forms in physiological processes, it is therefore essential to develop a spectroscopic procedure to understand this phenomenon. Electronic absorption (UV–vis) spectroscopy has proven effective in distinguishing the two forms^18^ and is sensitive to the number and nature of axially bound ligands.^7,46^ Nonetheless, to date, studies on this biomolecular switch through UV–vis spectroscopy have been strictly limited to the B_12_-cofactors^47^ or to **Cbl-1**.^48,49^ Beside absorption spectroscopy, further characterization of electronic transitions can be gained using electronic circular dichroism (ECD) and magnetic circular dichroism (MCD) spectroscopy. These techniques offer complementary insights, with MCD being particularly sensitive to metal coordination environments in metallo-organic complexes.^50^ In this contribution, we demonstrate that ECD and MCD spectroscopies supported by density functional theory (DFT) calculations and its time-dependent extension (TD-DFT) enable the determination of the molecular properties of photostable Cbl species in both base-on and base-off states.

To date, MCD spectra of both free Cbl molecules and enzyme-bound species have only been recorded at low temperatures (77 K) and in strong magnetic fields (7 T).^22,43,51–57^ In this work high-quality MCD spectra of β-axial and *meso*-modified Cbl species are obtained using 1.5 T static magnetic field, at room temperature, and with a relatively low number of accumulations.

## Experimental Section

### Materials

Vitamin B_12_, sodium hydroxide (NaOH), and 1 M hydrochloric acid solution (HCl) were purchased from Merck and used as received. Chemical derivatives were synthesized according to previously reported procedures.^1,27,58^

Cbl species in their base-on form were measured in aqueous solution (pH 7.4, 0.1 mg/mL). To obtain their base-off form, 0.1 mL of the aqueous stock solution (1 mg/mL) was taken and added to 0.9 mL of 1 M HCl solution to achieve a solution at 0.1 mg/mL concentration. Next, to revert to the former base-on form of Cbls, the appropriate volume of 18 M NaOH solution was added.

### Measurements

The UV–vis and ECD spectra of base-on **Cbl-s** in aqueous solution (c = 0.1 mg/mL) (see Figures S1 and S2) were recorded in 5 mm optical cells using a Jasco J-815SE spectropolarimeter. The parameters for the measurements were as follows: 10 scan accumulation, 200 nm/min scanning speed, 0.2 nm step size, 1 nm bandwidth, and a response time of 1 s. The spectral range for most compounds was from 220 to 650 nm, except for **Cbl-3**, which was measured up to 750 nm. All spectra were then background-corrected using the respective solvent measured under identical conditions.

MCD spectra were accumulated at room temperature using the Jasco J-815SE apparatus with a Jasco PMCD-586 permanent magnet, hosted in the optical sample compartment, which generates a magnetic field of ± 1.5 T. Since all compounds were chiral, obtaining MCD spectra required at least two ECD measurements, namely the two orientation of the magnetic field with respect to the incident radiation orientations. In order to check the presence of artifacts, we also measured an additional spectrum without magnetic field at the same instrumental conditions. The final MCD spectra (parallel and antiparallel) were derived by subtracting the zero-field spectrum from each and solvent contributions. To confirm the reproducibility of MCD spectra, additional measurements were conducted using a Jasco J-1500 spectrometer equipped with an MCD-581 electromagnet set to ± 1.5 T (Figure S3).

Additionally, the electronic spectra were recorded for **Cbl-1**, **Cbl-2**, **Cbl-4**, and **Cbl-6** in physiological conditions (pH 7.4), acidic conditions (≈ pH 1) (Figures S4–S10), and after adding NaOH solution to the acidic environment to induce the base-on form (Figures S6 and S9). In order to avoid incurring in possible sample decomposition, in this set of measurements, only five scans were used for both the base-on and base-off forms. Note, however, that after adding NaOH, it was not possible to obtain the base-on **Cbl-2** (Figure S10).

### Computational Details

Molecular systems were modeled starting from the most populated conformer already reported in the literature.^5^ Systems were then reoptimized at CAM-B3LYP/6–31G­(d)/MDF10 level of theory^59–61^ using the Gaussian16 suite of programs.^62^ The solvent effects (water) were accounted for using the PCM model.^63^ In order to simulate UV–vis and ECD spectra, different functionals were tested, the B3LYP providing the best agreement with experimental data (Figure S18). The base-off systems were modeled by starting from the corresponding Cbl geometry, which involved removing the phosphate-base loop and filling the cobalt coordination sphere with a water molecule. The UV–vis, ECD and MCD spectra were simulated using the ORCA 6.1.0 package^64,65^ in the TD-DFT framework^66^ at the same level of theory, taking into account the first 60 excited states. In view of the mixed nature of the electronic transitions in Cbls, we analyzed them using Natural Transition Orbitals (NTOs)^67^ instead of Kohn–Sham pairs, as NTOs provide a more intuitive and concise picture. NTO were generated using orca_plot utility and displayed with Jmol visualization software (isodensity surfaces at ± 0.04 e/bohr^3^).

## Results and Discussion

To investigate the conjugation of different functional groups to the *meso* position of the corrin ring (**Cbl-2**, **Cbl-3**, and **Cbl-6**), the effects of β-axial ligand exchange (**Cbl-4** and **Cbl-5**), and how various pH levels impact their electronic structure compared to **Cbl-1**, we selected five compounds. The molecular structures of the studied compounds are shown in Figure 1, together with a general structure of vitamin B_12_, to define terminology. Note that the detailed spectral behaviors of **Cbl-5** and **Cbl-6** are given in the Electronic Supporting Information.

The studied Cbl species, due to their vibrant colors, exhibit rich UV–vis, ECD, and MCD spectra, as shown in Figures 2, S1, and S2. Specifically, the UV–vis spectrum of **Cbl-1** shows the α, β, and γ bands at 550, 521, and 361 nm, respectively.^7,20^ Our TF-DFT simulations confirm, consistently with recent computational^5,58^ and experimental studies,^5,7,58,68,69^ that the α/β bands arise from the HOMO → LUMO electronic excitation and its vibronic progression, while the “*γ*-band-type” one is attributed mainly to the corrin π → π* transitions.^7,57^ Comparison of the absorption features reveals that the α/β spectral region is sensitive to *meso* modifications (i.e., **Cbl-2** and **Cbl-3**), whereas the γ band is more influenced by functionalization at the β-axial position (i.e., **Cbl-4** and **Cbl-5**).

**Figure 2:**
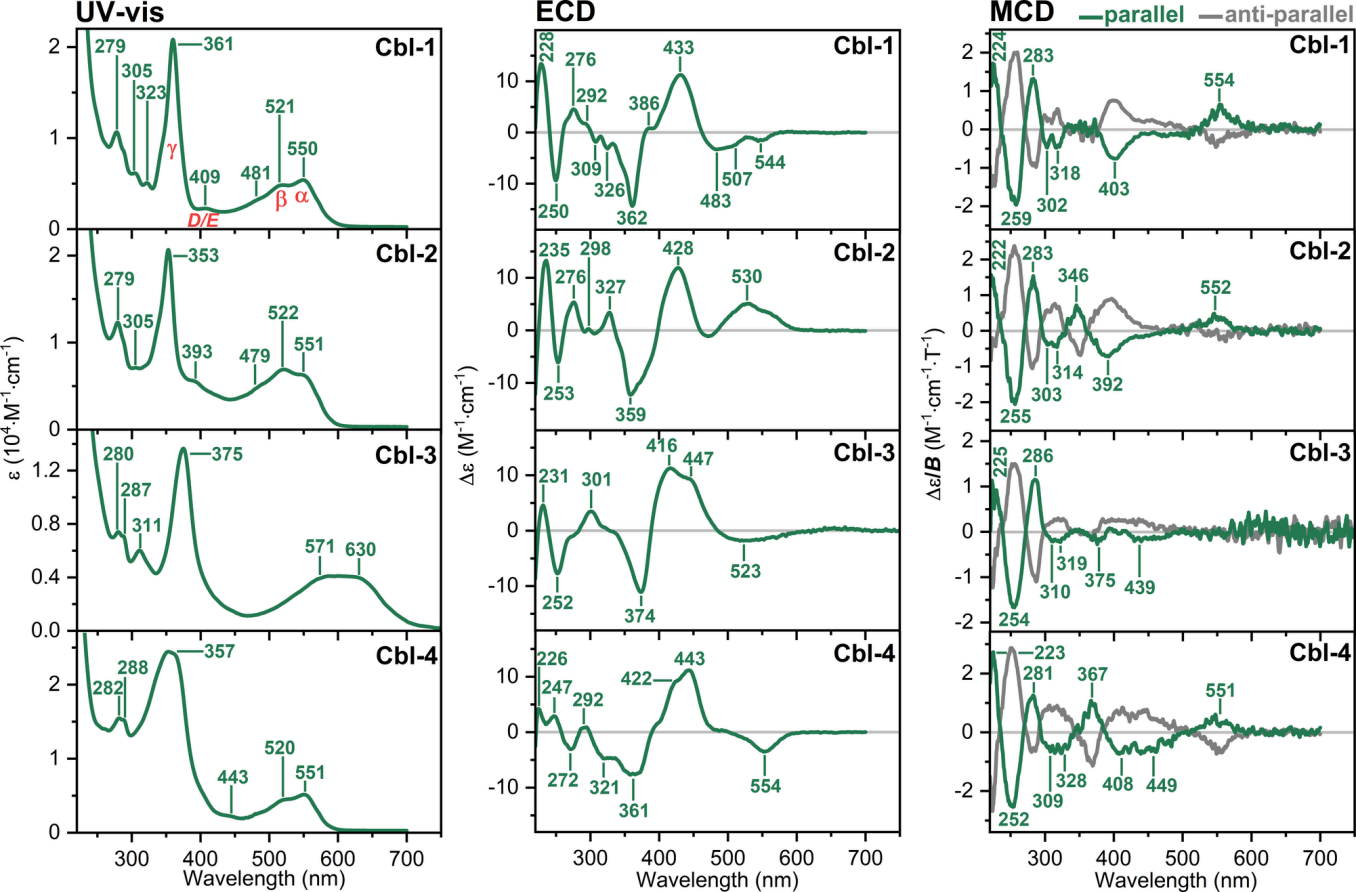
Experimental UV–vis, ECD, and MCD spectra of **Cbl-1** and three Cbl chemically modified in the base-on form (see the structures in Figure 1).

In fact, several differences appear in the UV–vis spectra when the CN ligand is replaced by an alkynyl moiety (i.e., 4-NO_2_–C_6_H_5_–C≡C in **Cbl-4** and C_6_H_5_–C≡C in **Cbl-5**). In aqueous medium, both Cbls species exhibit UV–vis spectra characterized by a prominent and broad γ feature at 360 nm and α/β bands at ≈ 550 and ≈ 520 nm, respectively, which is characteristic of alkynyl Cbls (Figures 2 and S2).^27^ Conversely, α/β electronic absorption profiles of *meso*-modified Cbls (**Cbl-2**, **Cbl-3**, and **Cbl-6**) depend on the electronic properties of the substituents. The NO_2_ and NH_2_ groups exhibit electron-withdrawing and electron-donating properties, respectively, resulting in hypsochromic and bathochromic shifts in the absorption spectra when compared to **Cbl-1**. Unlike the univocal behavior of NO_2_ and NH_2_ groups, bromine in **Cbl-6** exhibits dual electronic effects, acting both as a σ-withdrawing and a π-donor substituent (Figure S2).^46,70^ The π-donor effect seems to prevail, as indicated by the red shift in the electronic absorption of **Cbl-6** compared to **Cbl-1** (particularly in the α/β bands located in the visible region).

The ECD spectra, shown in Figure 2, demonstrate greater sensitivity to variations in the electronic structure of Cbls compared to UV–vis spectroscopy in characteristic features of electronic transitions, i.e., signs and intensities. The ECD profiles of *meso*-modified Cbls resemble that of nonmodified **Cbl-1**, characterized by a negative feature at ≈ 360 nm (γ region) and a broad, intense positive band between 400 and 450 nm (D/E region). Differences are most pronounced in the low-energy region, where substituents may lead to a reduction in the intensity of the first band associated with the corrin macrocycle. In the case of **Cbl-2**, even a sign inversion occurs. In contrast, the ECD spectra for β-axially modified Cbls show the α/β region to be more similar to that of **Cbl-1**. Only notable differences are observed in the γ region, where the negative band at ≈ 350 nm is significantly weaker in **Cbl-5** (Figure S2).

Although further refinements of the calculations could be made, since we considered only the most stable conformers and neglected vibronic effects, the electronic absorption and ECD spectra computed at the TD-DFT level successfully reproduced the experimentally observed trends (Figure S12). In the α/β region, these simulations allow for the assignment of transitions involving the metal center, which are not visible in UV–vis spectra because of their electric dipole-forbidden nature. Moreover, the first low-energy band of **Cbl-2** is correctly calculated as positive, whereas in all other cases, it is observed and calculated as negative. The ECD signal associated with the α band arises from an electric dipole transition moment oriented along the long axis of the corrin ring (Figure 1). In contrast, the magnetic dipole transition moment is almost perpendicular to the plane, and thus the ECD intensity is predicted to be small. It is then understandable that the presence of a polarizable NO_2_ group leads to a sign inversion of the ECD band compared to other Cbl derivatives.

Figure 2 reports also MCD spectra, which are equally sensitive to chemical modifications in the structure of the **Cbl-1** as the ECD signals. To our knowledge, the literature includes the MCD spectra of free (nonprotein-bound) **Cbl-1**.^7^ However, the spectroscopic signatures recorded previously for **Cbl-1** differ from those we report. In this study, we measured the MCD spectra of **Cbl-1** using two different spectrometers: one equipped with an electromagnet and the other with a permanent magnet, under similar experimental conditions. With the two experimental setup identical spectral patterns were recorded across the entire spectral region (Figure S3). The reported MCD spectra obtained by subtracting form the ECD spectra recorded in the presence of a parallel and antiparallel magnetic fields the intense ECD spectrum recorded at zero magnetic field, are mirror images of each other within experimental errors.^71,72^

There are several noticeable differences between the MCD spectra of **Cbl-1** and its modified analogs; however, the spectra remain similar in the high-energy range (220–330 nm). In this range, a characteristic −/+/−/+ patter of bands, going from high to low wavelengths, is observed when the measurements are taken under parallel magnetic field orientation (Figure 2). Next, interestingly, **Cbl-1** does not exhibit an MCD signal associated with the γ transitions, which instead appears in β-axially modified forms or when the NO_2_ group is introduced at the *meso* position. In turn, the α band is absent (within the sensitivity limits of our experimental setup) in the MCD spectra of **Cbl-3**, while for all the other compounds a weak positive MCD feature is measured.

To provide data useful for understanding the pH dependence of Cbl systems, the top panel of Figure 3 shows the UV–vis spectra of the **Cbl-1** and **Cbl-4** species measured in aqueous environment simulating physiological conditions (pH 7.4) and at pH 1. Similar to other Cbls provided in the Supporting Information, both compounds convert into their protonated base-off form in acidic environment. The detachment of the DmB moiety, followed by the replacement of the lower α-axial position by water molecules, leads to a hypsochromic shift in the α, β, and γ absorption bands with respect to the base-on form. Then, addition of saturated sodium hydroxide (NaOH, c = 18 M) solution to Cbls under acidic conditions forces them to revert to a stable base-on form, which is evident in their absorption spectra.

**Figure 3:**
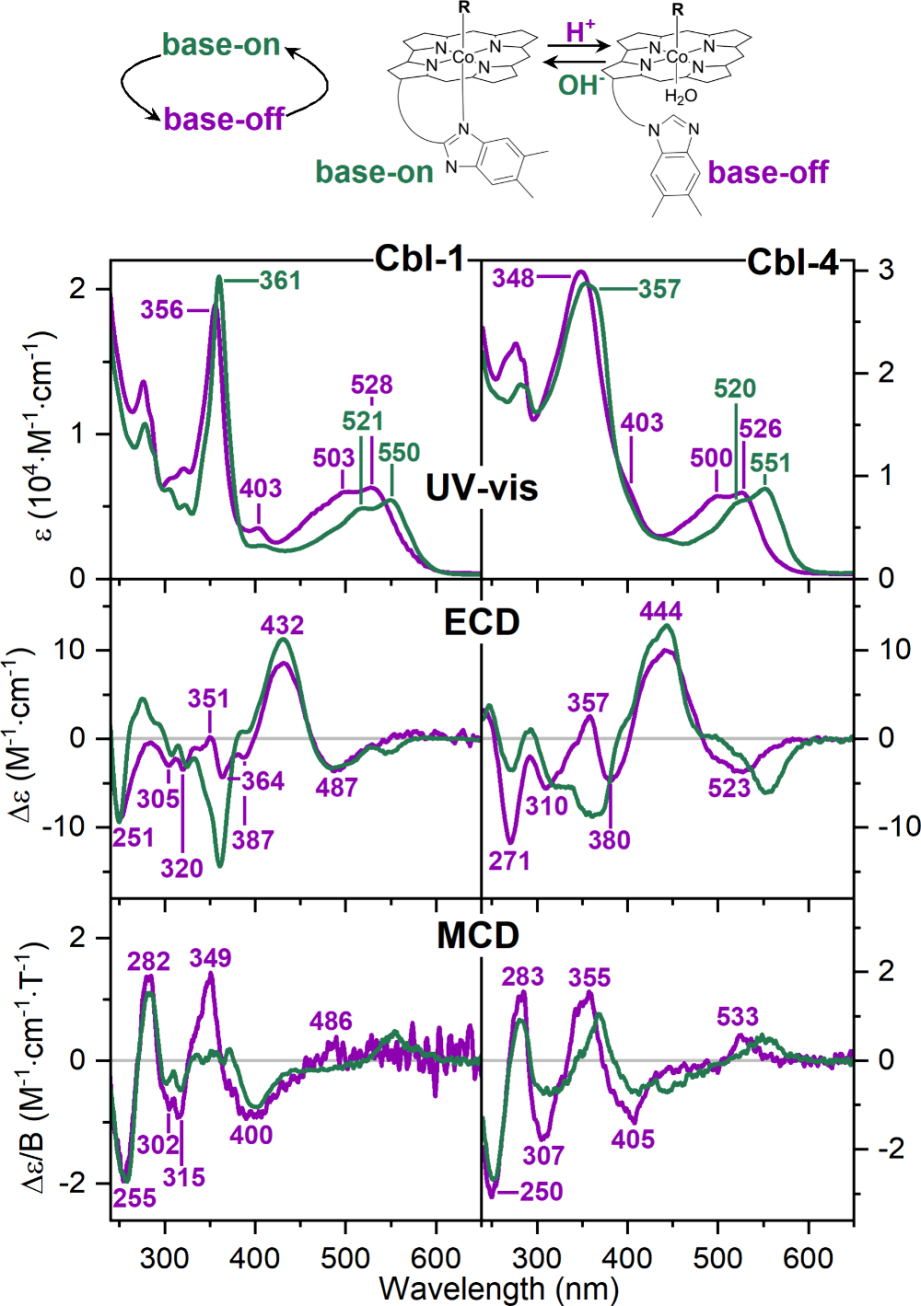
Experimental UV–vis, ECD, and MCD spectra of **Cbl-1** and **Cbl-4** measured in physiological (base-on) and acidic (base-off) conditions. MCD spectra are reported as the semidifference between parallel and antiparallel fields (see Figures S7 and S9 for each spectrum, see the structures in Figure 1).

Addition of hydrochloric acid (HCl, 1 M) to **Cbl-1** and **Cbl-4** aqueous solutions also results in characteristic ECD signals (middle panel of Figure 3). For native **Cbl-1**, the −/+/−/+ sign-pattern is observed at 487, 432, 364, and 351 nm. In contrast, **Cbl-4** displays signals at 523, 444, 380, and 357 nm, the most noticeable change being the sign change for the γ-band at 351 (**Cbl-1**) and 357 nm (**Cbl-4**). Also, the lower wavelength region of the ECD spectrum is significantly affected by pH, displaying negative features at 251 and 271 nm, respectively. When NaOH is subsequently added to the base-off system, the base-on forms are reformed, and the ECD spectra revert to the original state (Figures S6).

Replacing the DmB base with a water molecule alters the electronic structure of Cbl species, resulting in spectra related to those of the truncated vitamin B_12_ derivatives.^6^ Indeed, the band positions and signatures of the base-off **Cbl-1** and **Cbl-4** (Figure 3) closely match the previously published electronic spectra of the (CN)­(H_2_O)­Cby­(OMe)_7_ compound,^6^ in which water molecules occupy the DmB α-axial position (Figure S4). Shared features include the hypsochromic shift of the α, β, and γ absorption bands (with nearly identical intensities) and increased intensity, accompanied by frequency shift of the weak band designated as D/E toward shorter wavelengths. The blue-shifted absorption observed in the base-off form, compared to the base-on state, can be attributed to the elimination of σ-antibonding interactions, leading to a species-dependent stabilization of the HOMO relative to the LUMO orbital (Figures S12–S15).^9^ Additionally, the ECD spectra recorded for **Cbl-1** and **Cbl-4** are consistent with those obtained for (CN)­(H_2_O)­Cby­(OMe)_7_ compound across the entire spectral range, indeed suggesting that water molecules replace the DmB moiety in an acidic environment, where the base-off forms of Cbls occur.

Across all systems, both UV–vis and ECD spectra of the base-off forms exhibit similar trends (Figures 3 and S4). The most notable difference appears in the low-energy region, which resembles the patterns observed in the base-on forms and thus remains sensitive to structural changes. These findings suggest that either the β-axial ligand has limited influence on the electronic and geometric properties of Cbls in their base-off state, or that its effects are less pronounced than those resulting from the substitution of the DmB base with a water molecule. However, also in the γ region, a fairly intense positive ECD band between 350 and 360 nm is consistently observed across all base-off forms, making it a useful diagnostic marker for identifying base-off forms. So one may conclude that high-energy features (λ < 400 nm) are pH-sensitive, whereas low-energy ECD signals are substituent-sensitive and are not significantly affected by pH level. TD-DFT simulations of the base-on and base-off forms are reported in Figure 4 and Figures S12, S13. Despite some discrepancies–most notably in the high-energy region of the ECD spectrum, where the decision, imposed by system size constraints, to include only the most populated conformer and to limit the excited state number, likely plays a significant role, TD-DFT simulations provide a satisfactory reproduction of experimental data, effectively capturing the experimentally observed trends upon DmB removal. In particular, the emergence of the positive band in the γ region is correctly predicted.

**Figure 4:**
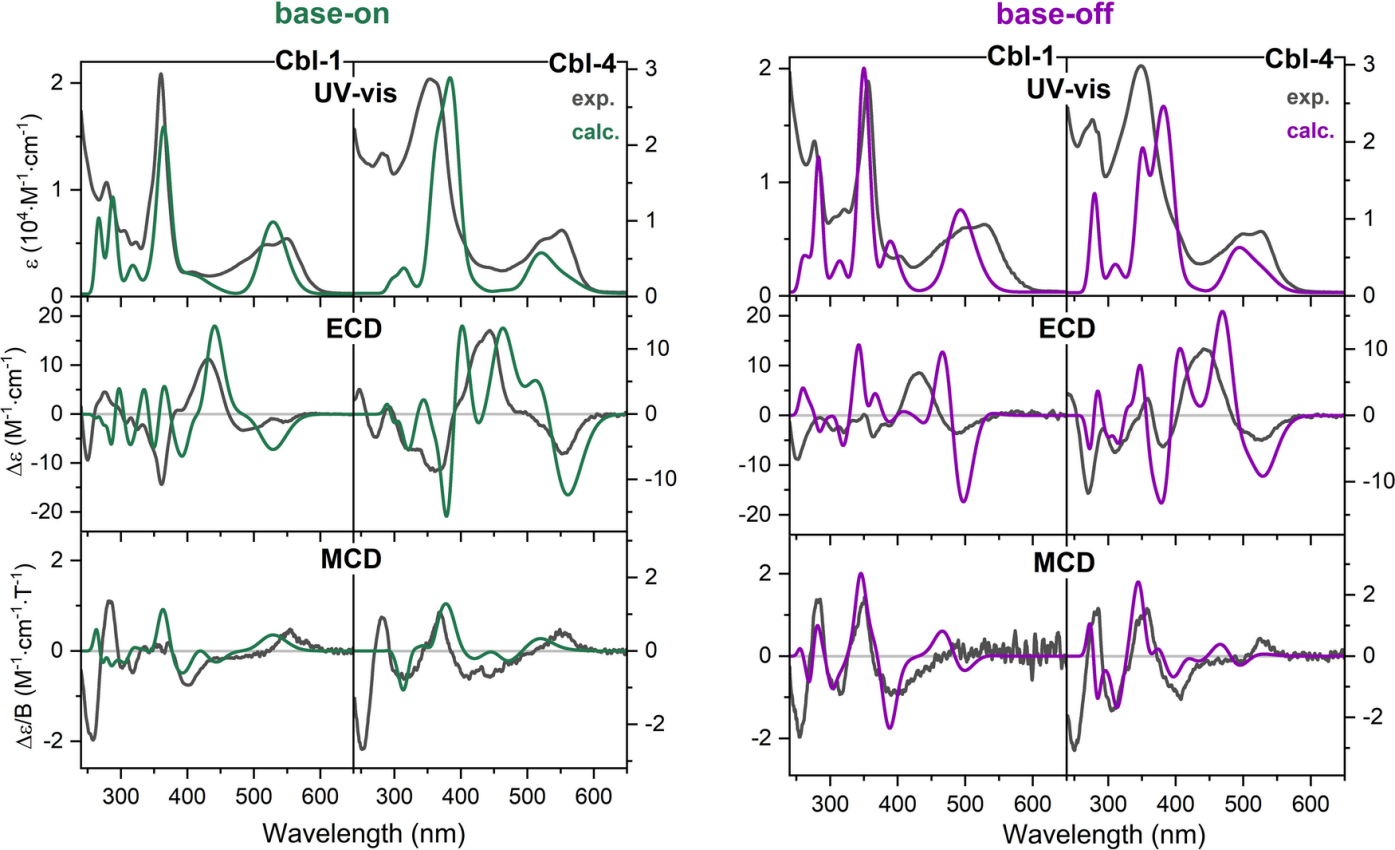
Comparison of experimental and simulated UV–vis, ECD, and MCD spectra of **Cbl-1** (left panel) and **Cbl-4** (right panel) in the base-on and base-off forms. Simulated spectra were convoluted with Gaussian functions (fwhm 1600 cm^–1^) and shifted in energy to match the experimental ones (1850 cm^–1^).

Although Cbls are generally capable of switching between the base-on and the base-off state, this molecular property can be limited at high concentrations and may vary with chemical modifications. Under strong acidic conditions, for example, **Cbl-1** and **Cbl-6** remain stable for about 6 h, while **Cbl-2** decomposes rapidly after acid addition. Notably, in this latter case, the base-on form could not be restored by adding NaOH (Figure S10).

We were intrigued by the detectable spectral changes observed in UV–vis and ECD signals after adding HCl to the aqueous solution of the native, β-axially, or *meso*-modified Cbls. We assumed that these variations would also be evident in the MCD spectra. Indeed, the experimental MCD spectra of the **Cbl-1** and **Cbl-4** compounds (lower panel of Figure 3) reveal several distinctive features resulting from the molecular switch that occurs under acidic conditions. These features include blue shifts of the MCD γ-band for **Cbl-4** (367 → 355 nm), the appearance of a new signal at 349 nm for **Cbl-1**, and the absence of a broad low-intensity shoulder at ≈ 450 nm for both native **Cbl-1** and **Cbl-4**. Moreover, the two MCD (base-on/base-off) spectra of **Cbl-1** confirm the shift of the positive α band to shorter wavelengths, which is observed at ≈ 485 nm in the base-off form and is no longer the first transition observed in absorption spectra from low energy. While in the absorption spectra the most evident difference among compounds with chemically different β-axial ligands lies in the shape of the γ-band, MCD ones show differences in the low-energy region of the spectra. The analysis of TD-DFT data and NTO pairs (Figures S14–S17) provides deeper insight into how MCD can discriminate among different β-axial substituents within the characteristic transition envelope generating the calculated bands, involving the metal and the β-axial ligand. Between 400 and 500 nm, both **Cbl-1** and **Cbl-4** exhibit two negative MCD signals (403 nm plus a weak shoulder for **Cbl-1**, 408 and 449 nm for **Cbl-4**). However, their intensity ratios differ significantly and are characteristic of the specific axial ligand (see also Figure S1). MCD spectroscopy also reflects more similar spectral profiles among the base-off forms, with the most appreciable difference appearing in the lowest-energy transition. The characteristic signature observed in MCD spectra allows classification of a Cbl spectrum as belonging to the base-off form, even for compounds with similar absorption spectra (see, for instance, the **Cbl-2** base-on and **Cbl-1** base-off absorption spectra). Furthermore, in agreement with previous results,^73^ the resonance Raman (RR) spectral signatures indicate that the formal oxidation state remains +3 in both base-on and base-off forms (see description and Figure S11).

## Conclusions

In summary, we studied the biomolecular switch whereby the DmB base is either coordinated (base-on) or dissociated (base-off) from the cobalt center across a series of Cbls modified at the corrin ring and at the upper β-axial ligand. We found that this switch-type behavior may be selectively controlled by pH; clear-cut signals of how Cbls switch were successfully obtained through ECD and MCD. The sensitivity of the electronic spectral methods is evident in different band shapes and positions, and disappearance of some signals. This allowed identification of marker bands characteristic of the base-off form, which can be used to monitor the biologically important molecular switch in Cbl species. Furthermore, we presented reproducible MCD spectra acquired with equipment readily accessible in most spectroscopic laboratories. The approach presented here can be used to investigate Cbls in solutions at concentrations standard at physiological conditions and has potential for studying complex (bio)­systems.

## Supplementary Material



## Notes

UV–vis and ECD spectra in the presence of the two orientations of the magnetic field of all derivatives; base-on and base-off UV–vis, ECD and MCD spectra; experimental procedure and Raman spectra; TD-DFT simulated spectra and NTO graphical representations (PDF)

## ∇

Department of Biochemistry, University of Colorado, Boulder, CO 80309–0596, USA; BioFrontiers Institute, University of Colorado, Boulder, CO 80303–0596, USA
