# Supplementary material for: Monitoring the Switching from Base-on to Base-off Forms of Vitamin B12 by Natural and Magnetic Circular Dichroism Spectroscopies
Source: Anal Chem. 2026 Jan 30;98(5):3466–73. doi: 10.1021/acs.analchem.5c07584 (PMC12903063; doi:10.1021/acs.analchem.5c07584)
Supplement: Supplementary file 1 [file ac5c07584_si_001.pdf]

# Supporting Information

## Monitoring the Switching from Base-on to Base-off Forms of Vitamin $B_{12}$ by Natural and Magnetic Circular Dichroism Spectroscopies

Ewa Machalska,[*,†,‡] Giuseppe Mazzeo,[†] Aleksandra J. Wierzba,[¶,#] Jakub Dybaś,[‡] Joanna E. Rode,[§] Sergio Abbate,[†,||] Dorota Gryko,[*,¶] Malgorzata Baranska,[⊥] Giovanna Longhi,[*,†,||] and Marco Fusè[*,†]

[†] *Department of Molecular and Translational Medicine, Università di Brescia, 25123 Brescia, Italy*

[‡] *Jagiellonian Centre for Experimental Therapeutics (JCET), Jagiellonian University, 30-348 Krakow, Poland*

[¶] *Institute of Organic Chemistry, Polish Academy of Sciences, 01-224 Warsaw, Poland*

[§] *Laboratory for Spectroscopy, Molecular Modeling and Structure Determination, Institute of Nuclear Chemistry and Technology, 03-195 Warsaw, Poland*

[||] *Istituto Nazionale di Ottica CNR, Unità di Brescia, Via Branze 45, 25123 Brescia, Italy*

[⊥] *Faculty of Chemistry, Jagiellonian University, 30-387 Krakow, Poland*

[#] Current address: Department of Biochemistry, University of Colorado, Boulder, CO 80309-0596, USA; BioFrontiers Institute, University of Colorado, Boulder, CO 80303-0596, USA

E-mail: ewa.machalska@unibs.it; dorota.gryko@icho.edu.pl; giovanna.longhi@unibs.it; marco.fuse@unibs.it

## TABLE OF CONTENTS

**Figure S1.** Comparison of experimental UV-vis, ECD, and MCD spectra of base-on Cbls. **S3**

**Figure S2.** Experimental UV-vis, ECD, and MCD spectra of **Cbl-5** and **Cbl-6** in the base-on form. **S4**

**Figure S3.** Comparison of experimental MCD spectra of base-on **Cbl-1** in aqueous solution measured using two different spectrometers and magnets. **S4**

**Figure S4.** Experimental UV-vis and ECD spectra of **Cbl-1**, **Cbl-2**, **Cbl-4**, and **Cbl-6** measured under physiological (base-on) and acidic (base-off) conditions, compared to the electronic spectra of heptamethyl cobyrinate species. **S5**

**Figure S5.** Experimental UV-vis and ECD spectra of **Cbl-4** measured under physiological (base-on) and acidic (base-off) conditions. **S6**

**Figure S6.** UV-vis and ECD spectra of **Cbl-4** and **Cbl-6** measured in physiological (base-on) and acidic (base-off) conditions. **S6**

**Figure S7.** Experimental MCD spectra of **Cbl-1** measured in physiological (base-on) and acidic (base-off) conditions. **S7**

**Figure S8.** Experimental MCD spectra of **Cbl-2** measured in physiological (base-on) and acidic (base-off) conditions. **S7**

**Figure S9.** Experimental MCD spectra of **Cbl-4** measured in physiological (base-on) and acidic (base-off) conditions, and after adding NaOH solution. **S8**

**Figure S10.** Experimental UV-vis and ECD spectra of **Cbl-2** measured in physiological (base-on) and acidic (base-off) conditions, and after adding NaOH solution. **S9**

**Figure S11.** RR spectra of **Cbl-4** measured in physiological (base-on) and acidic (base-off) conditions. **S10**

**Figure S12.** Comparison of experimental and calculated UV-vis and ECD spectra of native **Cbl-1** and its analogs in the base-on form. **S11**

**Figure S13.** Calculated UV-vis, ECD, and MCD spectra of Cbls species in their base-on and base-off forms. **S12**

**Figure S14.** Graphical representation of the Natural Transition Orbitals of the first three transitions in **Cbl-1** and the base-off model system. **S13**

**Figure S15.** Graphical representation of the Natural Transition Orbitals of transitions from the $4^{th}$ to the $6^{th}$ in **Cbl-1** and the base-off model system. **S14**

**Figure S16.** Graphical representation of the Natural Transition Orbitals of transitions from the $7^{th}$ to the $9^{th}$ in **Cbl-1** and the base-off model system. **S15**

**Figure S17.** Graphical representation of the Natural Transition Orbitals of transitions from the $10^{th}$ to the $12^{th}$ in **Cbl-1** and the base-off model system. **S16**

**Figure S18.** Comparison of experimental and simulated UV-vis, ECD, and MCD spectra of **Cbl-1**. **S17**

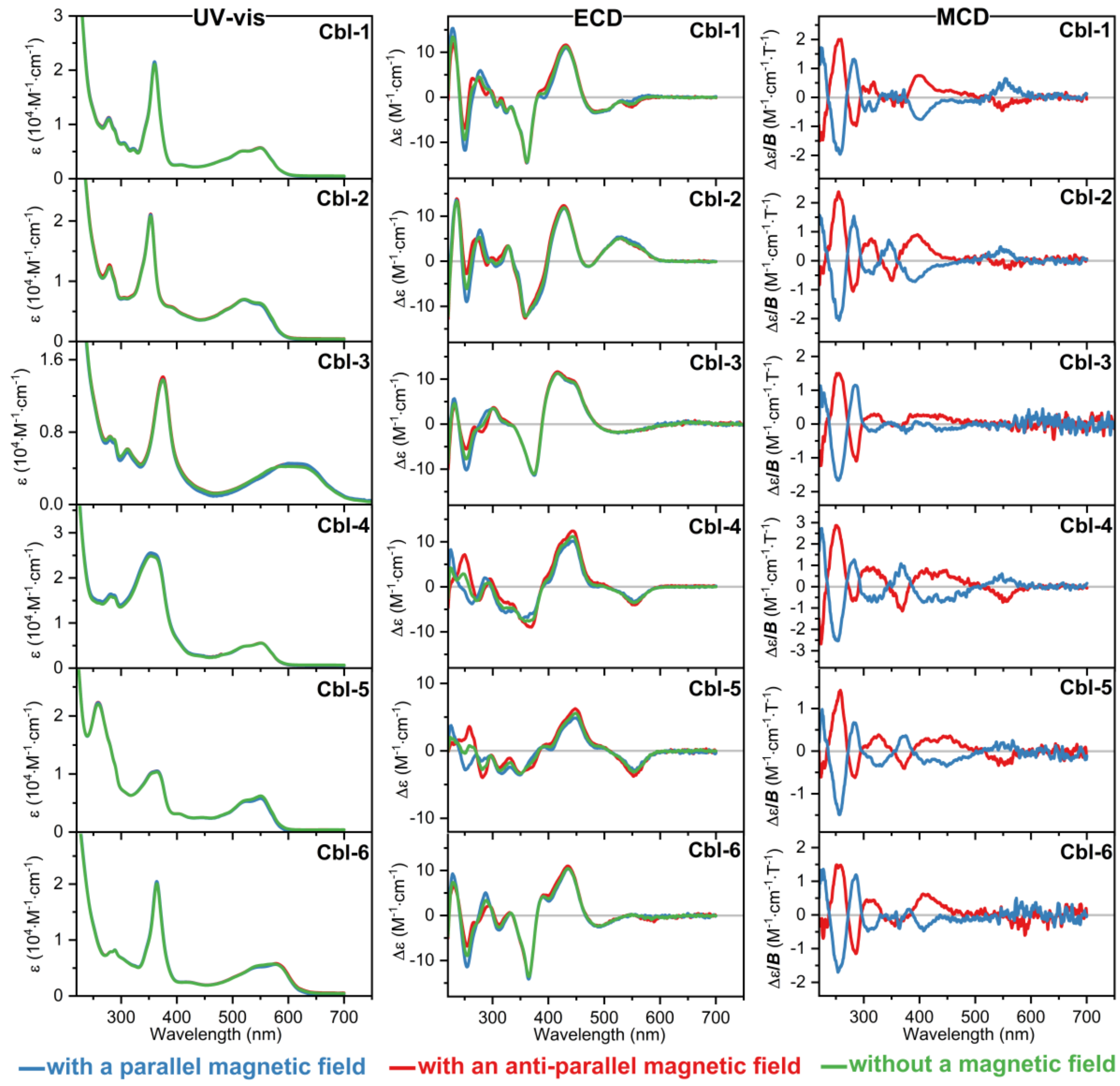


**Figure S1.** Comparison of experimental UV-vis, ECD, and MCD spectra of base-on Cbls in aqueous solution measured with (blue and red lines) or without (green line) a magnetic field. See the main text for the procedure for obtaining the MCD spectra of chiral molecules.

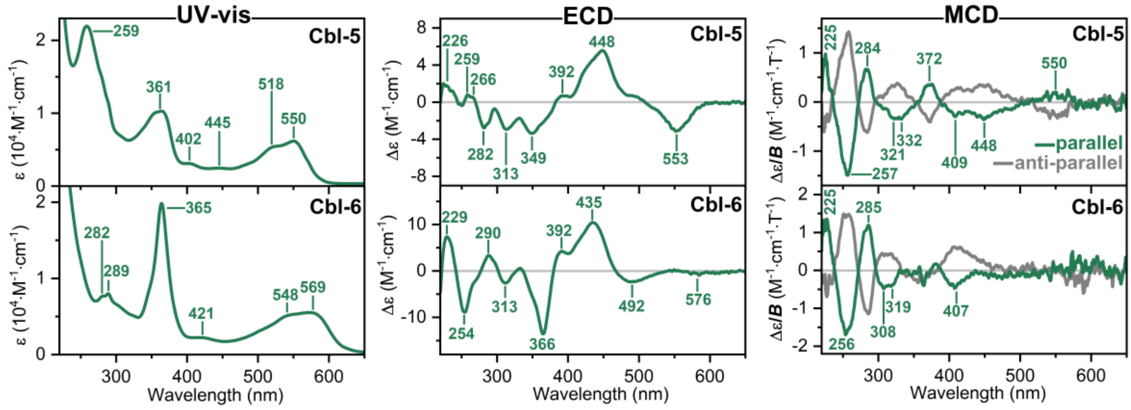


**Figure S2.** Experimental UV-vis, ECD, and MCD spectra of **Cbl-5** and **Cbl-6** in the base-on form.

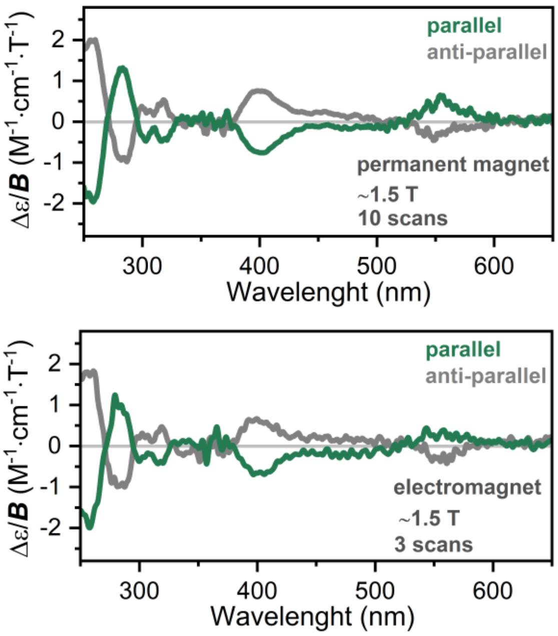


**Figure S3.** Comparison of experimental MCD spectra of base-on **Cbl-1** in aqueous solution measured using two different spectrometers and magnets (upper spectrum: Jasco J-815SE with a Jasco PMCD-586 permanent magnet; bottom spectrum: Jasco J-1500 with an MCD-581 electromagnet).

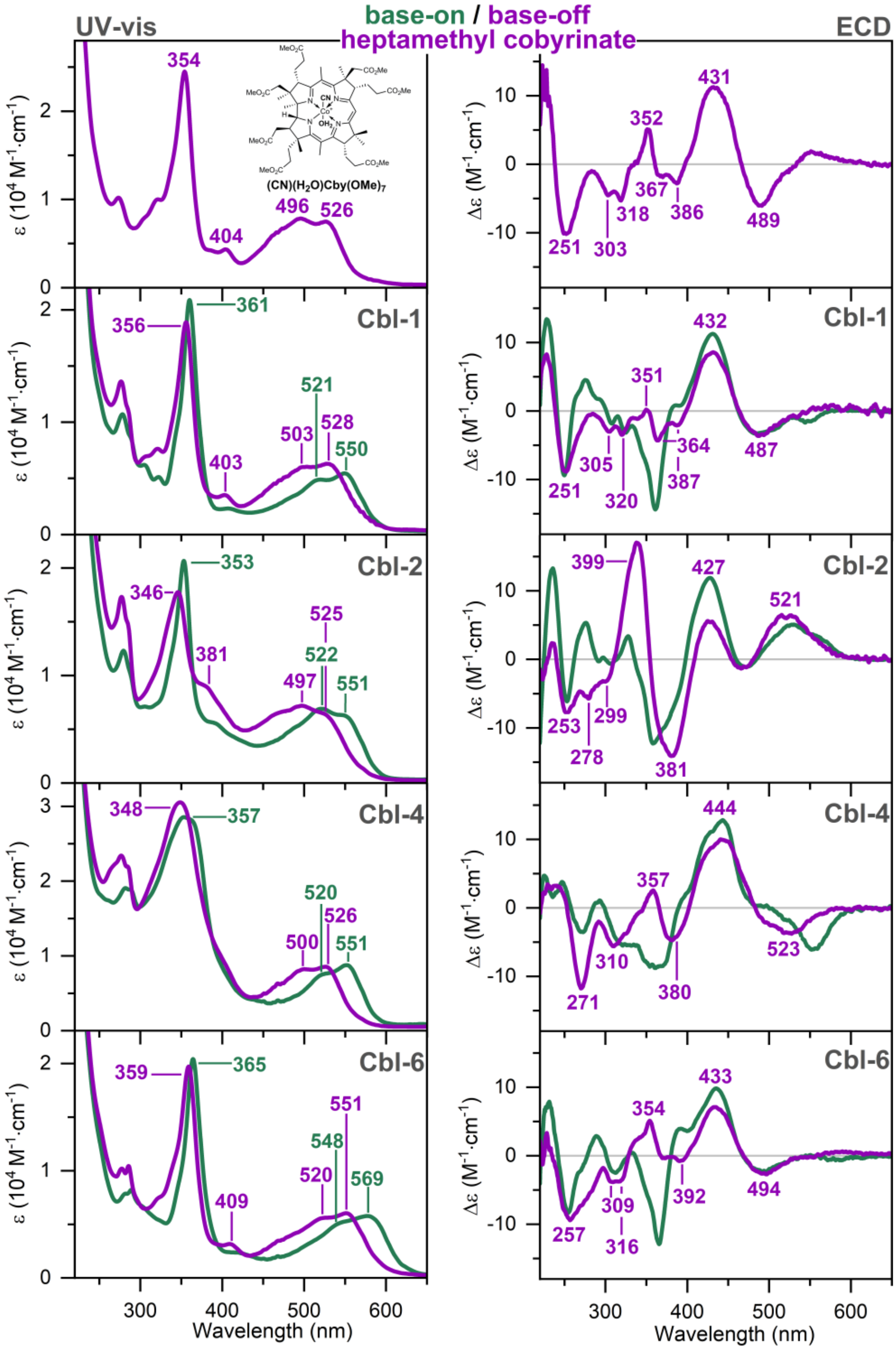


**Figure S4.** Experimental UV-vis and ECD spectra of **Cbl-1**, **Cbl-2**, **Cbl-4**, and **Cbl-6** measured under physiological (base-on) and acidic (base-off) conditions, compared to the electronic spectra of heptamethyl cobyrinate ((CN)($H_2O$)Cby(OMe)$_7$) species. The data concerning UV-vis and ECD spectra of ((CN)($H_2O$)Cby(OMe)$_7$) are reused with permission from an already published paper.[4]

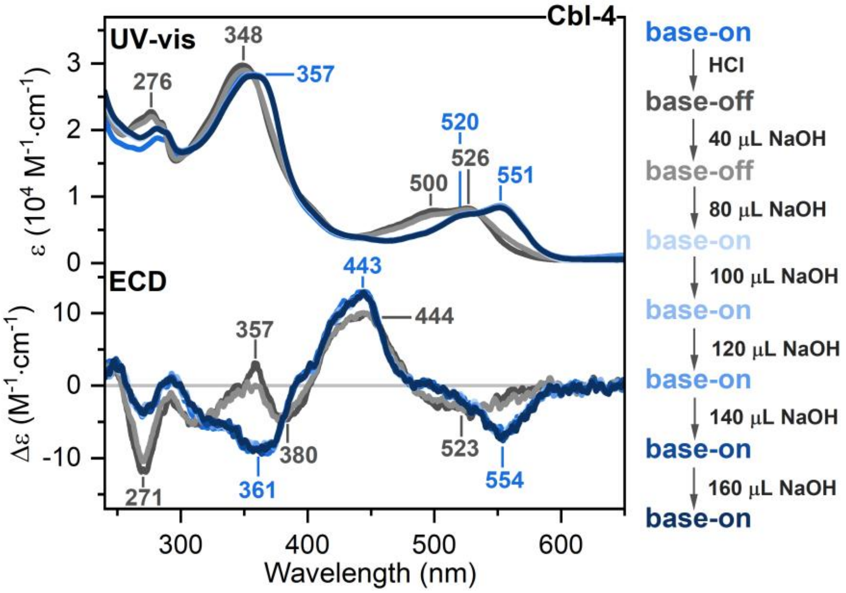


**Figure S5.** Experimental UV-vis (upper panel) and ECD (lower panel) spectra of **Cbl-4** measured under physiological (base-on) and acidic (base-off) conditions. To activate the base-on state of **Cbl-4**, 40, 80, 100, 120, 140, and 160 μL of NaOH solution were added to the acidic solution, where **Cbl-4** exists as the base-off form.

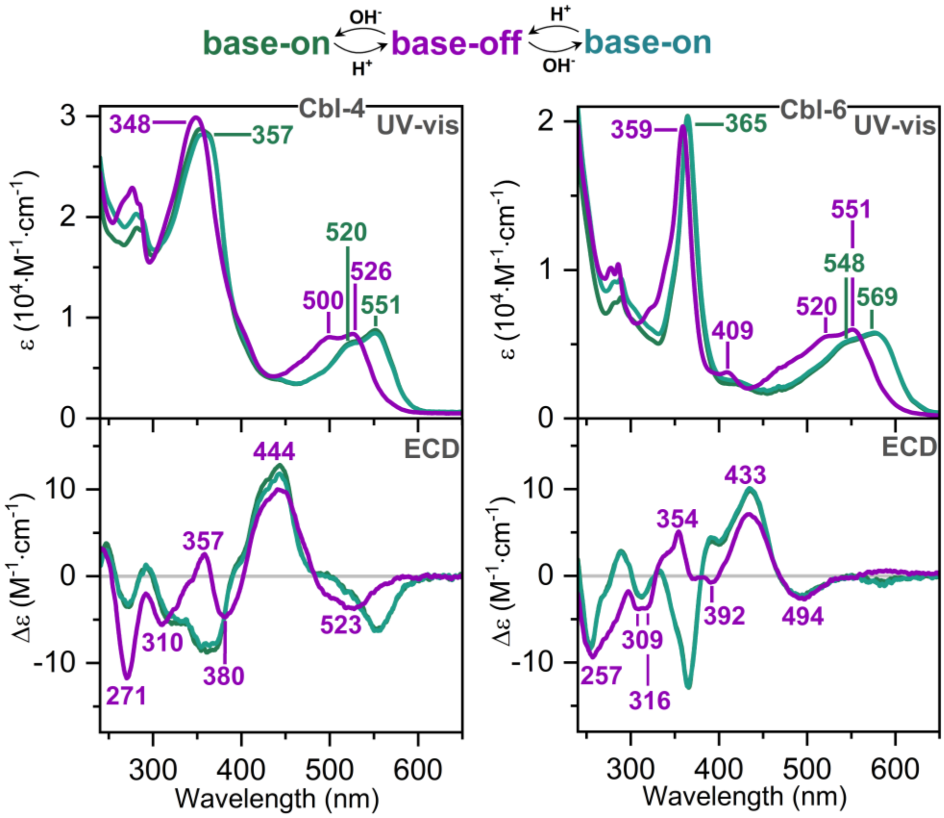


**Figure S6.** UV-vis and ECD spectra of **Cbl-4** and **Cbl-6** measured in physiological (base-on) and acidic (base-off) conditions, and after adding 80 μL (**Cbl-4**) and 60 μL (**Cbl-6**) solution of NaOH to the acidic environment to induce base-on form.

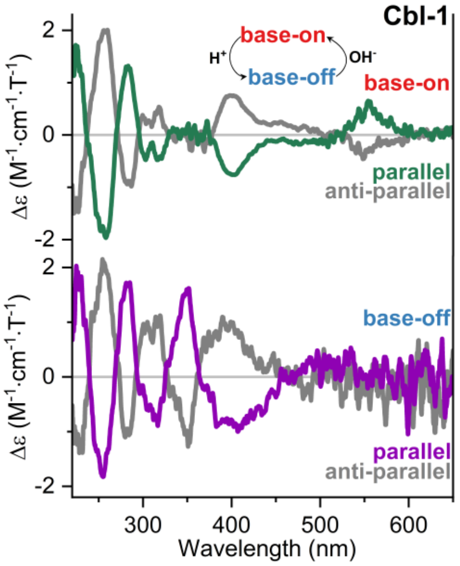


**Figure S7.** Experimental MCD spectra of **Cbl-1** measured in physiological (base-on) and acidic (base-off) conditions.

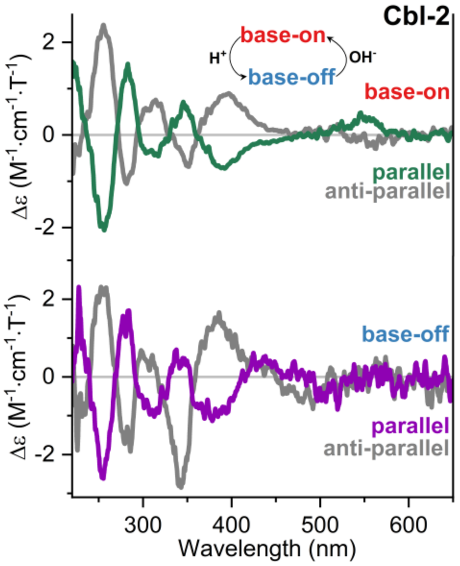


**Figure S8.** Experimental MCD spectra of **Cbl-2** measured in physiological (base-on) and acidic (base-off) conditions.

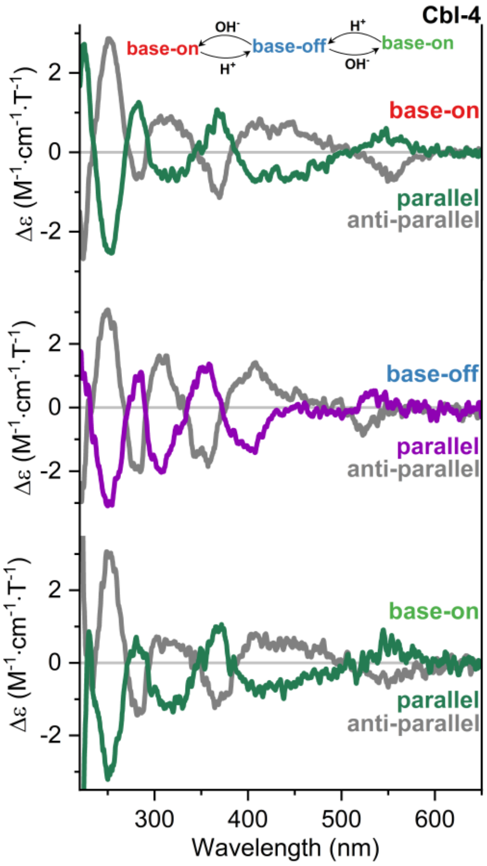


**Figure S9.** Experimental MCD spectra of **Cbl-4** measured in physiological (base-on) and acidic (base-off) conditions, and after adding 65 μL solution of NaOH to the acidic environment to induce base-on form.

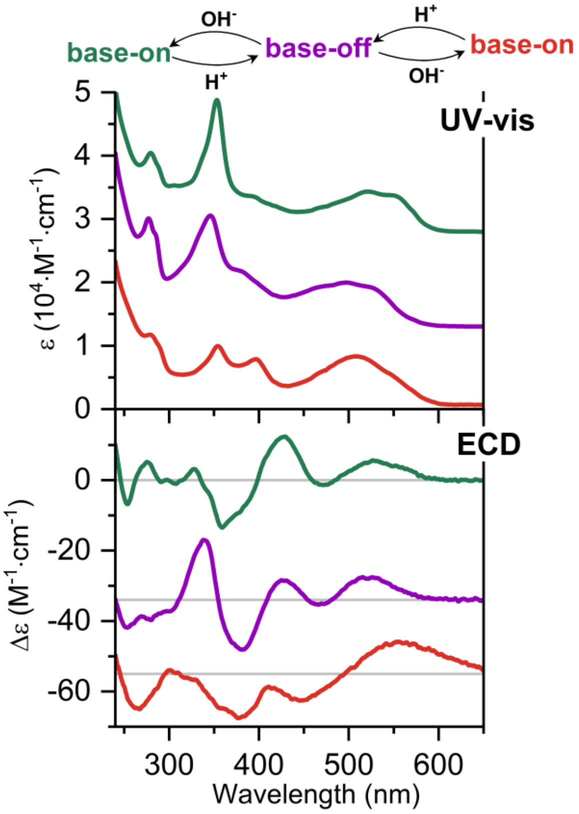


**Figure S10.** Experimental UV-vis (upper panel) and ECD (lower panel) spectra of **Cbl-2** measured in physiological (base-on) and acidic (base-off) conditions, and after adding 80 µL of NaOH solution to the acidic environment to induce the base-on form. Note, however, that it was impossible to obtain the base-on **Cbl-2** form after adding NaOH.

***Resonance Raman (RR) measurements***

RR spectra of **Cbl-4** (**Figure S11**, **panel A**) in base-on state were recorded using a *Chiral*RAMAN-2X$^{TM}$ spectrometer (BioTools Inc.) equipped with an excitation wavelength of 532 nm, within the 2500–250 cm$^{-1}$ spectral range, with a resolution of 7 cm$^{-1}$, concentration of 0.1 mg/mL, laser power of 200 mW, and an integration time of 4 s.

On the other hand, the RR spectra of **Cbl-4** (**Figure S11**, **panels B-E**) in both states (base-on and base-off) and at a concentration of 1 mg/mL were recorded using a WITec confocal alpha300 RSA + Raman microscope (WITec GmbH, Ulm, Germany). The applied excitation wavelength was 405 nm, sourced from an air-cooled diode laser, and the Raman scattering was detected by the back-illuminated Andor Newton 970 CCD camera (Oxford Instruments, Abingdon, England) cooled to −60° C. Spectra were collected using the dry Nikon S Plan Fluor objective (20×/ 0.45NA). The laser power at the sample was adjusted accordingly to avoid CCD camera saturation and overheating of the sample in the measured spot, varying between 2 and 5 mW. The spectra represent an average of at least 30 accumulations with 1 s exposure time, collected from three randomly chosen spots within the sample.

Considering resonance Raman spectra, the vibrational signals of **Cbl-4** species at ~1500 and 1595 cm$^{-1}$ (obtained for laser excitation at 405 nm) are more affected than other bands and may serve as signatures of the molecular switch caused by HCl addition (150 µL). The addition of acid to the alkaline environment induces a change in the relative intensities of these bands. The higher-intensity band (~1500 cm$^{-1}$) is attributed to the long-axis-polarized corrin macrocycle vibrations and is assigned to the in-phase C=C and C=N stretching modes along

the longer axis of the corrin ring. Instead, the RR band at the higher frequency (~1595 $cm^{-1}$) is primarily assigned to C=C and C=N stretching modes in the corrin macrocycle.[3,5]

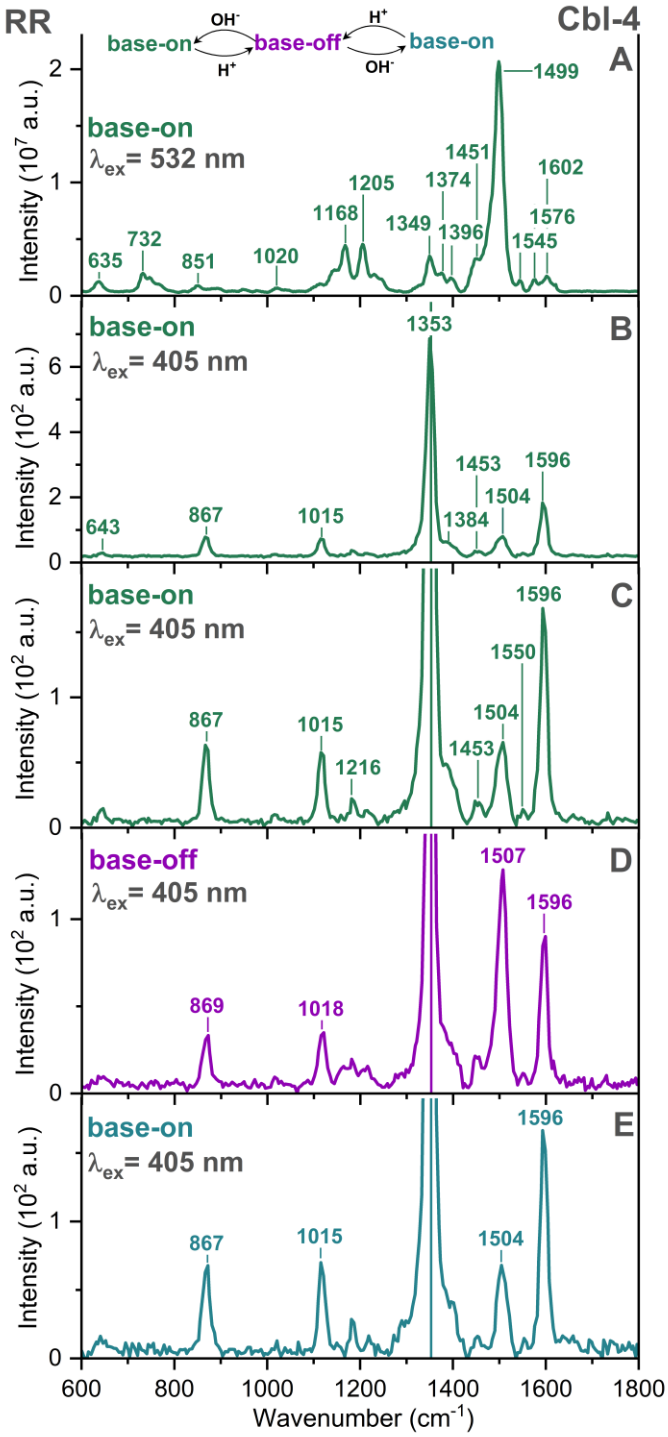


**Figure S11.** RR spectra of **Cbl-4** measured in physiological (base-on) and acidic (base-off) conditions using 532 nm (**panel A**) and 405 nm (**panels B**, **C**, **D**, and **E**) excitation laser lines. Panels **C** and **D** show the RR spectra of the base-on and base-off forms. Panel **E** displays the Raman spectrum in the base-on state formed after adding NaOH solution. The differences in the relative intensities between the RR spectra collected with 405 and 532 nm excitations are due to differences in resonance conditions.

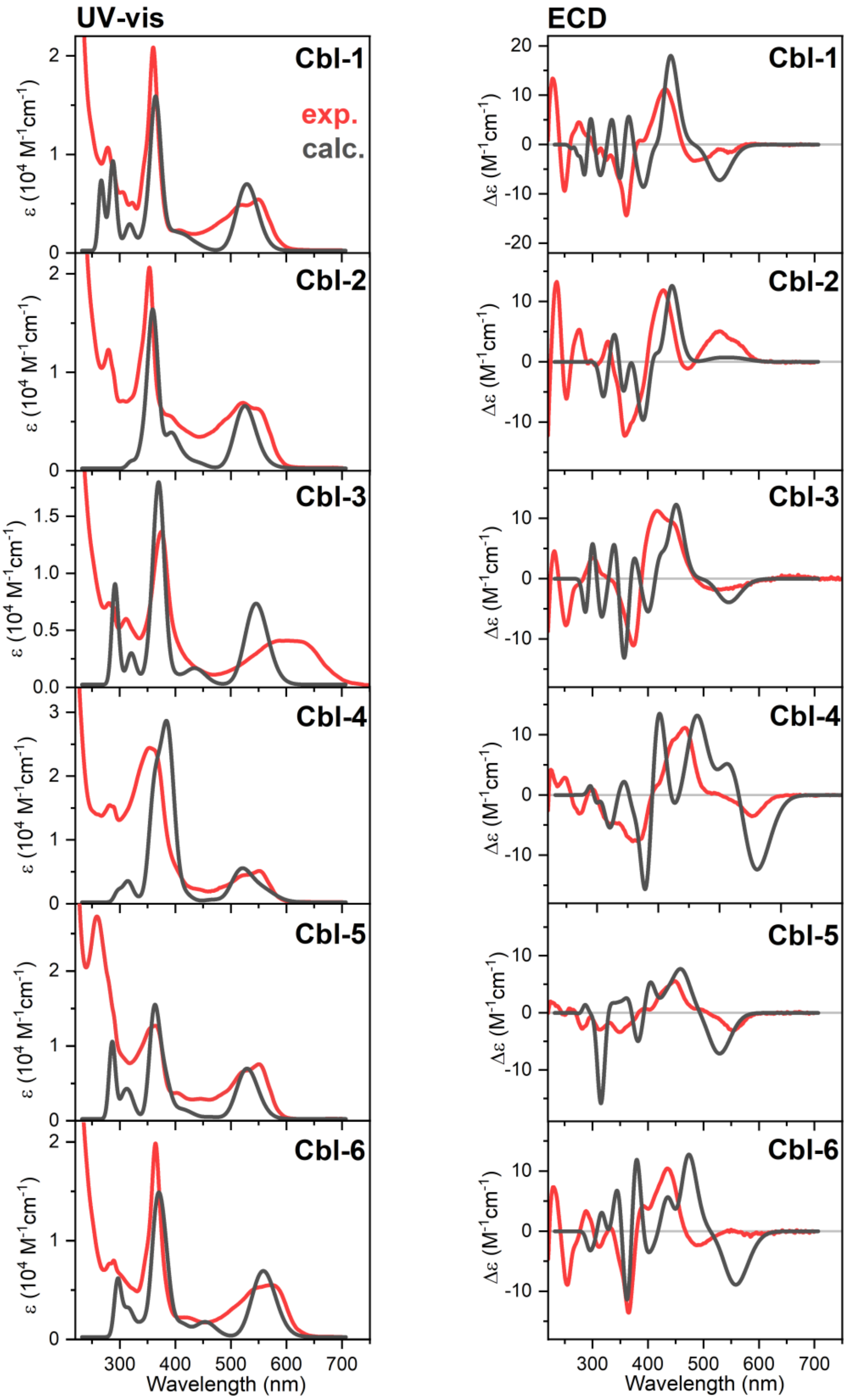


**Figure S12.** Comparison of experimental (red line) and calculated (black line) UV-vis and ECD spectra of native **Cbl-1** and its analogs in the base-on form. The spectra were calculated at the B3LYP/6-31G(d)/MDF10/PCM level of theory. Simulated spectra were convoluted with Gaussian functions (FWHM 1500 $cm^{-1}$) and shifted in energy to match the experimental ones (2000 $cm^{-1}$).

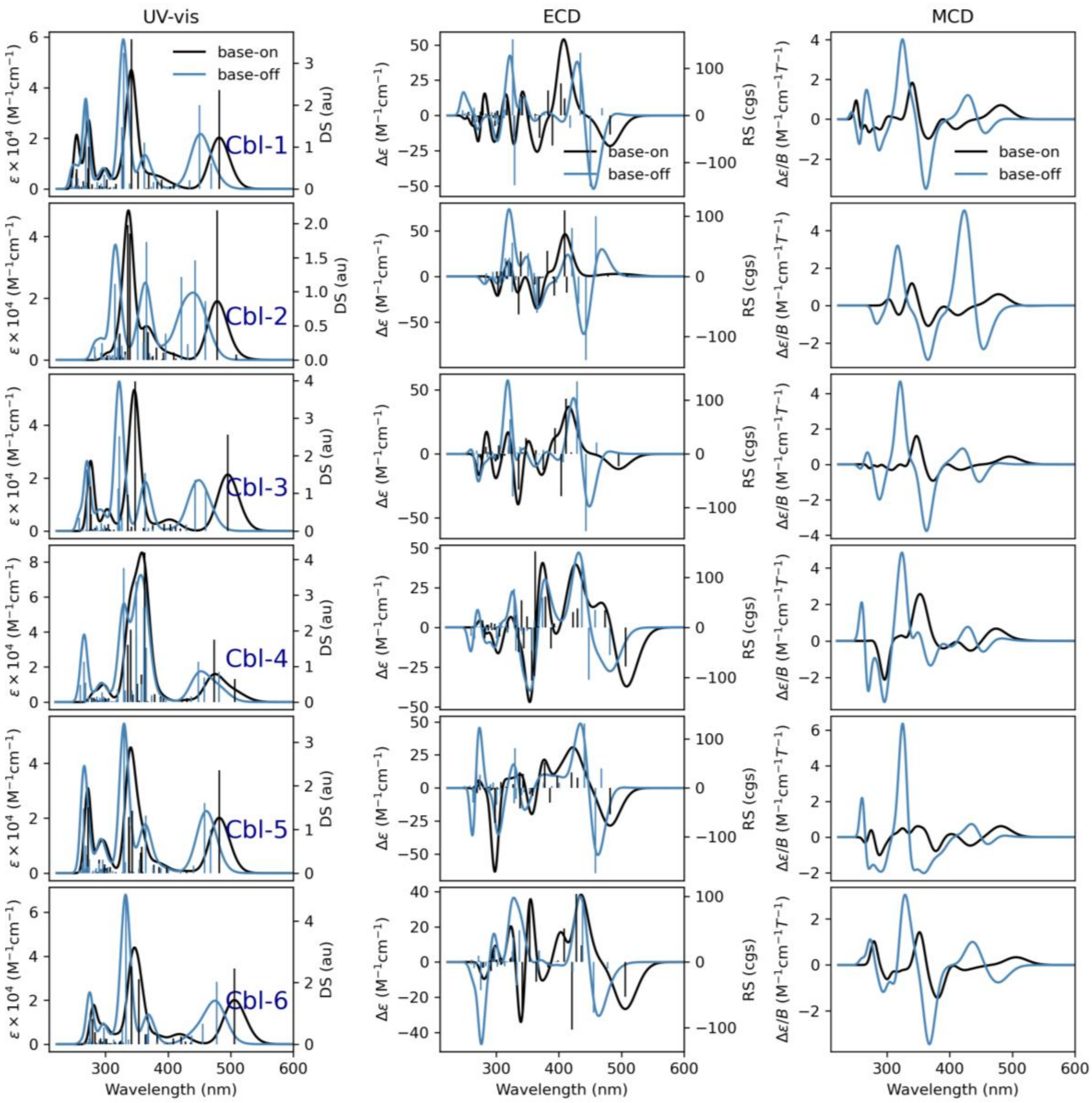


**Figure S13.** Calculated UV-vis, ECD, and MCD spectra of base-on (black line) and base-off (blue line) of Cbl species. The spectra were calculated using B3LYP/6-31G(d)/MDF10/PCM level of theory. Simulated spectra were convoluted with Gaussian functions (FWHM 1500 $cm^{-1}$, left axis). For each spectrum, dipole and rotational strengths of the transitions are also reported as sticks (cgs units, right axis).

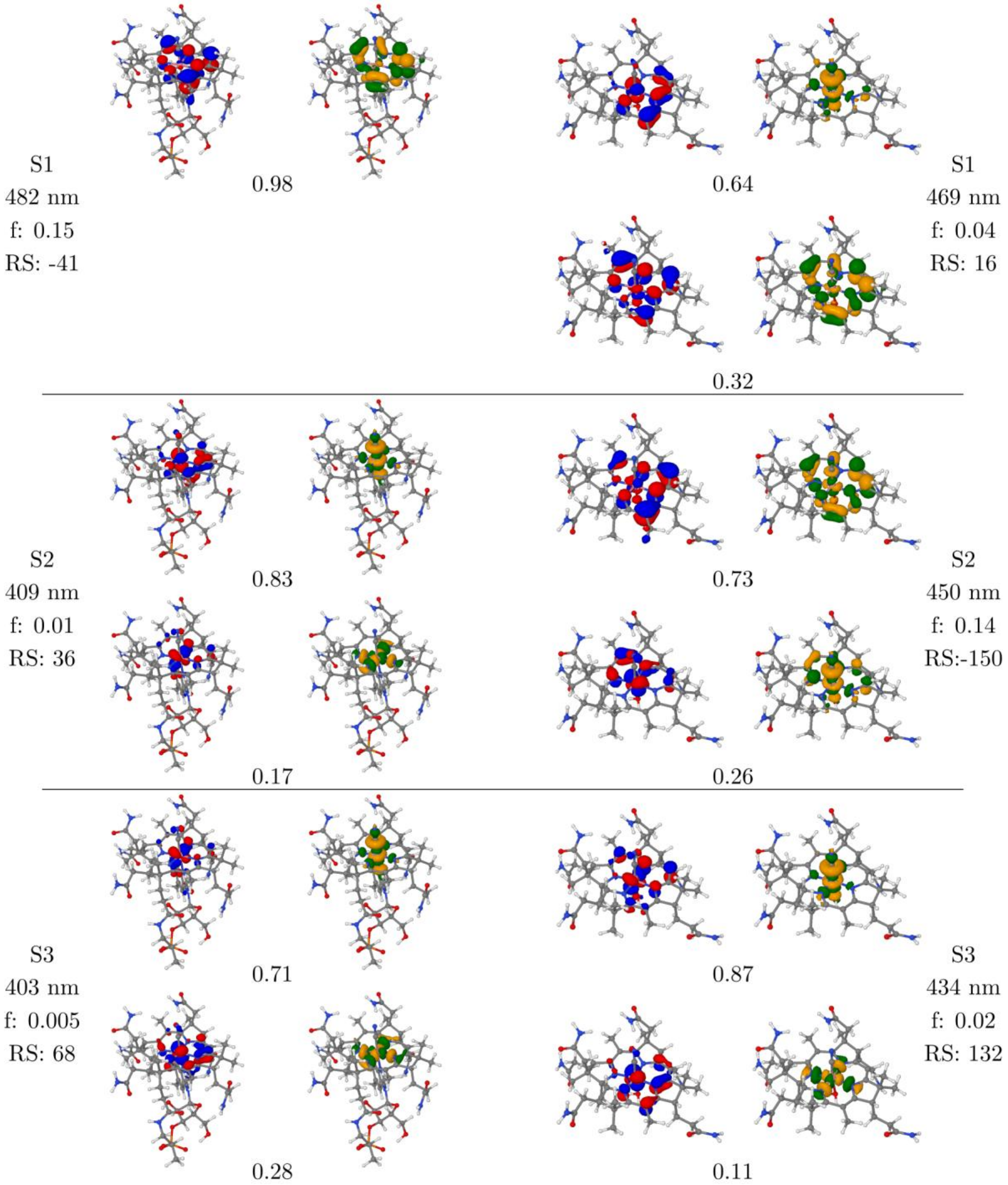


**Figure S14.** Graphical representation of the Natural Transition Orbitals of the first three transitions in **Cbl-1** (on the left) and the base-off model system (on the right). Only occupied (holes, depicted as red-blue isosurfaces) and unoccupied (electrons, orange-green isosurfaces) NTO pairs that contribute more than 95% to each excited state are displayed. The wavelengths in nm, oscillator strengths (f), and rotational strengths (RS) of the transitions are also provided.

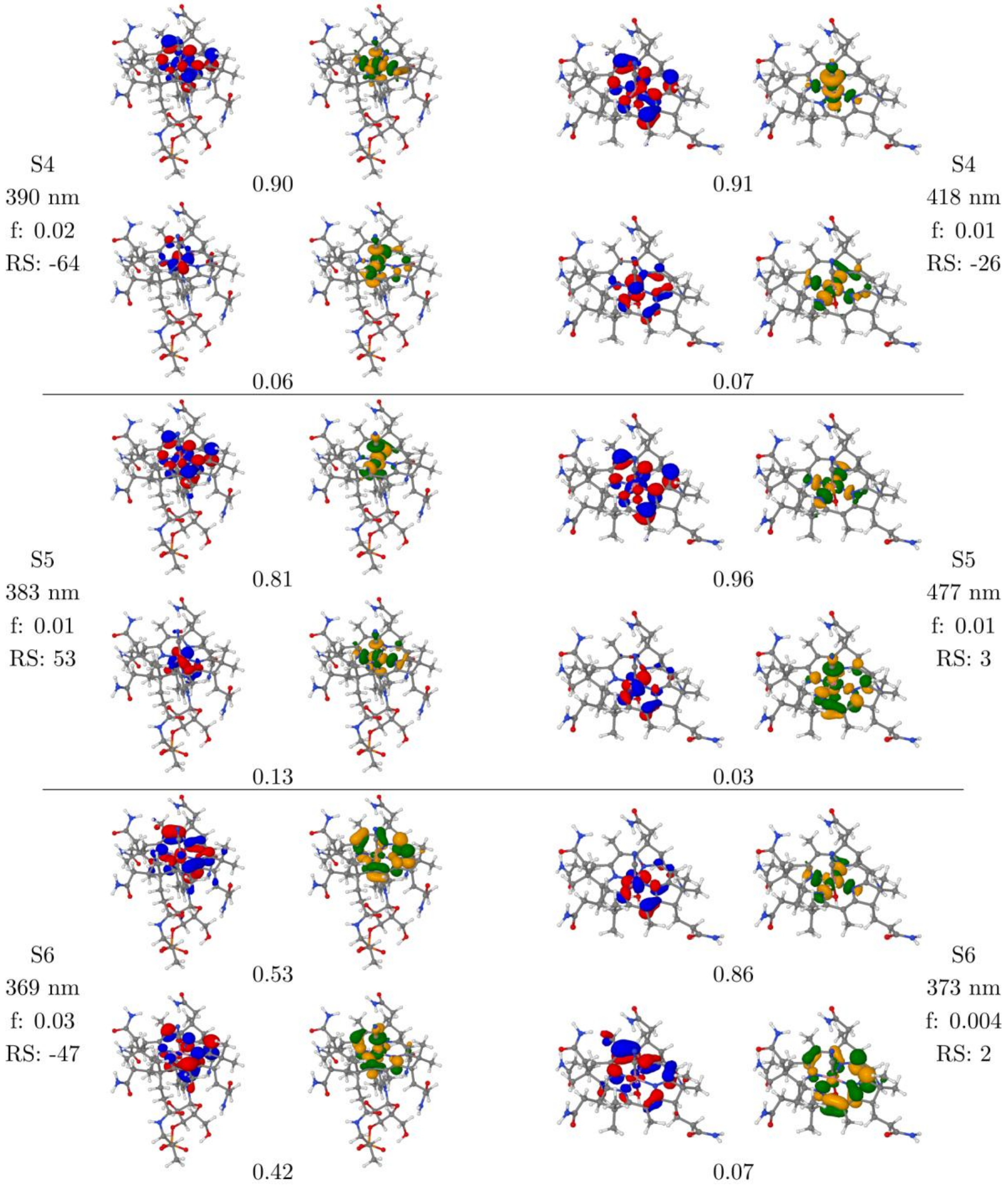


**Figure S15.** Graphical representation of the Natural Transition Orbitals of transitions from the $4^{th}$ to the $6^{th}$ in **Cbl-1** (on the left) and the base-off model system (on the right). Only occupied (holes, shown as red-blue isosurfaces) and unoccupied (electrons, orange-green isosurfaces) NTO pairs that contribute more than 90% to each excited state are displayed. The wavelengths in nm, oscillator strengths (f), and rotational strengths (RS) of the transitions are also reported.

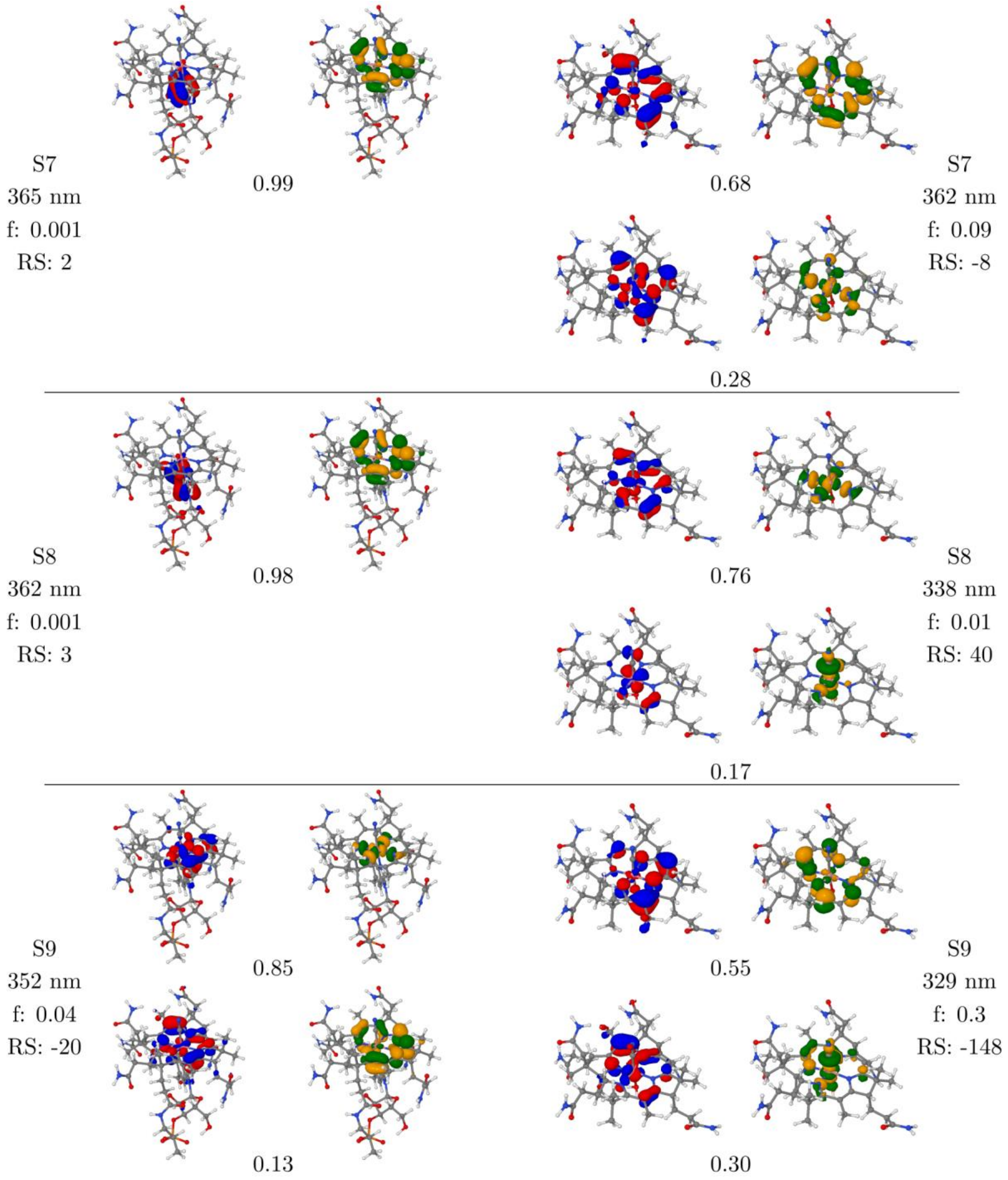


**Figure S16.** Graphical representation of the Natural Transition Orbitals of transitions from the 7$^{th}$ to the 9$^{th}$ in **Cbl-1** (on the left) and the base-off model system (on the right). Only occupied (holes, shown as red-blue isosurfaces) and unoccupied (electrons, orange-green isosurfaces) NTO pairs that contribute more than 95% to each excited state are displayed. The wavelengths in nm, oscillator strengths (f), and rotational strengths (RS) of the transitions are also reported.

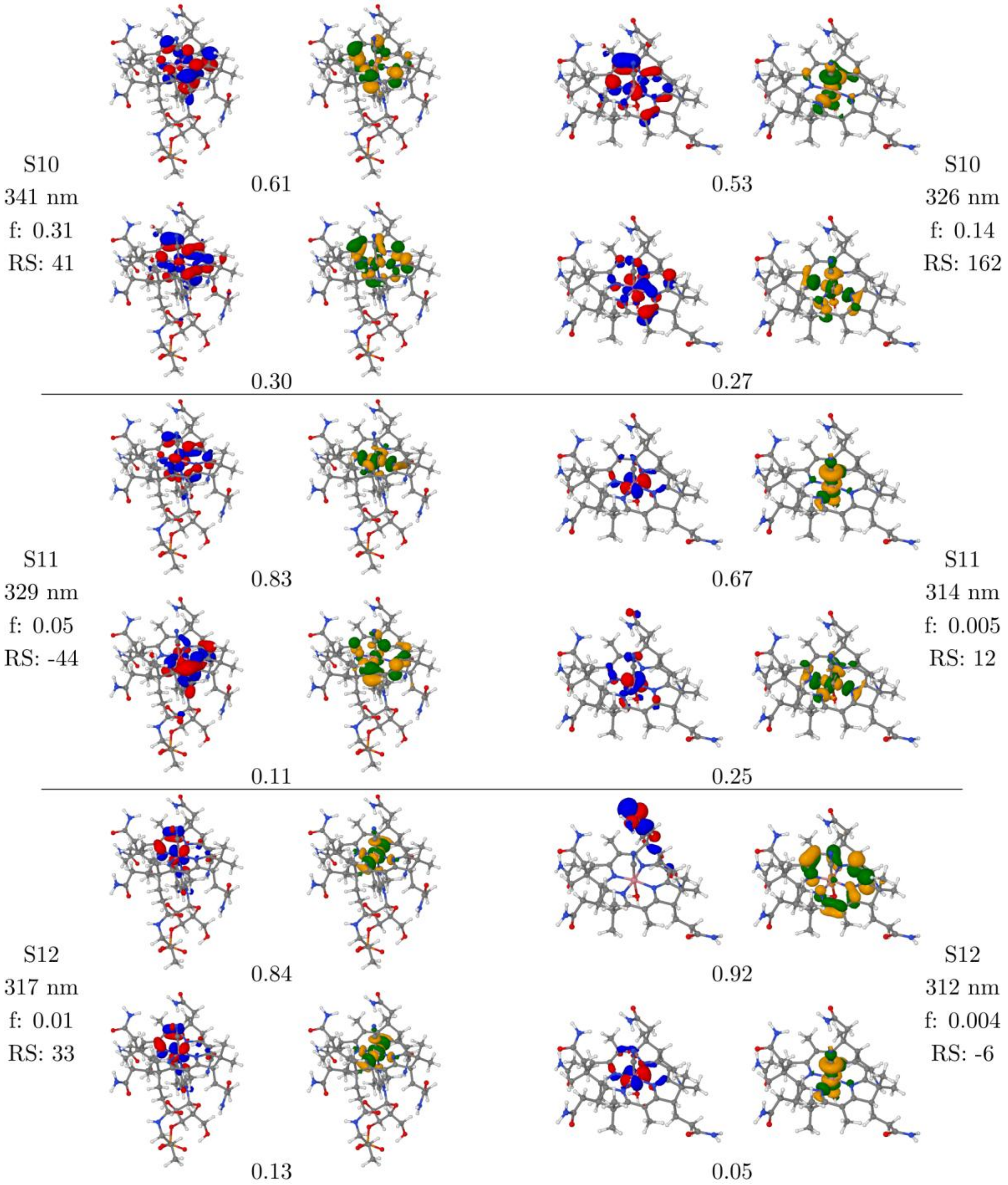


**Figure S17.** Graphical representation of the Natural Transition Orbitals of transitions from the $10^{th}$ to the $12^{th}$ in **Cbl-1** (on the left) and the base-off model system (on the right). Only occupied (holes, shown as red-blue isosurfaces) and unoccupied (electrons, orange-green isosurfaces) NTO pairs that contribute more than 95% to each excited state are displayed. The wavelengths in nm, oscillator strengths (f), and rotational strengths (RS) of the transitions are also reported.

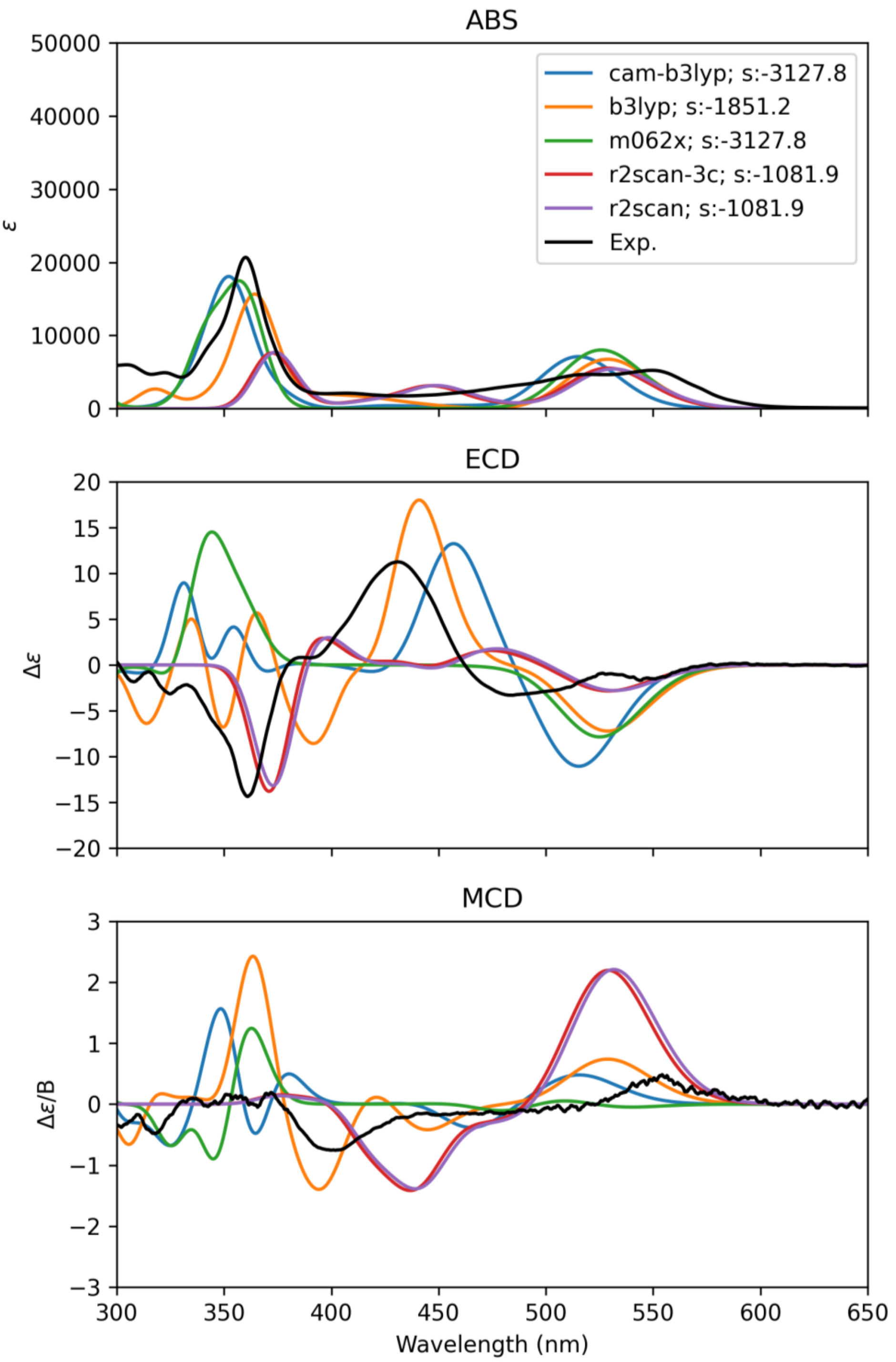


**Figure S18.** Comparison of experimental and simulated UV-vis, ECD, and MCD spectra of **Cbl-1**. In the caption the applied shift in $cm^{-1}$ applied to the different functionals is reported. Simulated spectra were convoluted with Gaussian functions (FWHM 1600 $cm^{-1}$).
